# The French national protocol for Kennedy’s disease (SBMA): consensus diagnostic and management recommendations

**DOI:** 10.1186/s13023-020-01366-z

**Published:** 2020-04-10

**Authors:** Pierre-François Pradat, Emilien Bernard, Philippe Corcia, Philippe Couratier, Christel Jublanc, Giorgia Querin, Capucine Morélot Panzini, François Salachas, Christophe Vial, Karim Wahbi, Peter Bede, Claude Desnuelle, on behalf of the French Kennedy’s Disease Writing Group, Nadine Le Forestier, Andoni Echaniz-Laguna, Giorgia Querin, Gianni Sorarù, Thierry Perez, Cédric Ramos, Cyril Goizet, Jean Claude Desport, Michel Pugeat, Bertrand Pichon, Sandrine Maniez, Julie Robillard, Christophe Coupe, Laurence Laurier Betram, Sandra Roy Bellina, Nathalie Lévêque, Jérôme Penot, Valérie Goutines Caramel

**Affiliations:** 1 AP-HP, Groupe Hospitalier Universitaire APHP-Sorbonne Université, site Pitié-Salpêtrière, Département de Neurologie, Centre de Reference pour la SLA et les Maladies du Motoneurone, CNRS, INSERM, Laboratoire d’Imagerie Biomédicale, 47 Boulevard de l’Hôpital - F-75634, 13 Paris Cedex, France; 2 Service d’électromyographie et pathologies neuromusculaire, Centre de Reference pour la SLA et les Maladies du Motoneurone, CHU Lyon Hôpital Neurologique P. Wertheimer, Lyon, France; 3 Département de Neurologie, Centre de Reference pour la SLA et les Maladies du Motoneurone, CHU Tours, Tours, France; 4 Département de Neurologie, Centre de Reference pour la SLA et les Maladies du Motoneurone, CHU Limoges, Limoges, France; 5 AP-HP, Groupe Hospitalier Universitaire APHP-Sorbonne Université, site Pitié-Salpêtrière Département d’endocrinologie, Paris, France; 6 AP-HP, Groupe Hospitalier Universitaire APHP-Sorbonne Université, site Pitié-Salpêtrière, Service de Pneumologie, Médecine Intensive et Réanimation (Département R3S), F-75013 Paris, France; 7 Sorbonne Université, INSERM, UMRS1158 Neurophysiologie Respiratoire Expérimentale et Clinique, F-75005 Paris, France; 8 AP-HP, Groupe Hospitalier Universitaire APHP-Sorbonne Université, site Pitié-Salpêtrière, Département de Neurologie, Centre de Reference pour la SLA et les Maladies du Motoneurone, Paris, France; 9 Service d’électromyographie et pathologies neuromusculaire, Centre de Reference pour la SLA et les Maladies du Motoneurone, CHU Lyon Hôpital Neurologique P. Wertheimer, Lyon, France; 10 AP-HP, CHU Cochin, Service de cardiologie, Centre de référence des maladies neuromusculaires Nord/Est/Ile de France, Paris, France; 11 Computational Neuroimaging Group, Trinity College Dublin, Dublin, Ireland; 12 Pôle Neurosciences - Service de Neurologie, Centre de Référence SLA et les maladies du neurone moteur, Hôpital Pasteur2, CHU de Nice Université Côte d’Azur, Nice, France

**Keywords:** Kennedy disease, Spinal and bulbar muscular atrophy, Guidelines, Polyglutamine, Androgen receptor, Androgen insensitivity

## Abstract

**Background:**

Kennedy’s disease (KD), also known as spinal and bulbar muscular atrophy (SBMA), is a rare, adult-onset, X-linked recessive neuromuscular disease caused by CAG expansions in exon 1 of the androgen receptor gene (AR). The objective of the French national diagnostic and management protocol is to provide evidence-based best practice recommendations and outline an optimised care pathway for patients with KD, based on a systematic literature review and consensus multidisciplinary observations.

**Results:**

The initial evaluation, confirmation of the diagnosis, and management should ideally take place in a tertiary referral centre for motor neuron diseases, and involve an experienced multidisciplinary team of neurologists, endocrinologists, cardiologists and allied healthcare professionals. The diagnosis should be suspected in an adult male presenting with slowly progressive lower motor neuron symptoms, typically affecting the lower limbs at onset. Bulbar involvement (dysarthria and dysphagia) is often a later manifestation of the disease. Gynecomastia is not a constant feature, but is suggestive of a suspected diagnosis, which is further supported by electromyography showing diffuse motor neuron involvement often with asymptomatic sensory changes. A suspected diagnosis is confirmed by genetic testing. The multidisciplinary assessment should ascertain extra-neurological involvement such as cardiac repolarisation abnormalities (Brugada syndrome), signs of androgen resistance, genitourinary abnormalities, endocrine and metabolic changes (glucose intolerance, hyperlipidemia). In the absence of effective disease modifying therapies, the mainstay of management is symptomatic support using rehabilitation strategies (physiotherapy and speech therapy). Nutritional evaluation by an expert dietician is essential, and enteral nutrition (gastrostomy) may be required. Respiratory management centres on the detection and treatment of bronchial obstructions, as well as screening for aspiration pneumonia (chest physiotherapy, drainage, positioning, breath stacking, mechanical insufflation-exsufflation, cough assist machnie, antibiotics). Non-invasive mechanical ventilation is seldom needed. Symptomatic pharmaceutical therapy includes pain management, endocrine and metabolic interventions. There is no evidence for androgen substitution therapy.

**Conclusion:**

The French national Kennedy’s disease protocol provides management recommendations for patients with KD. In a low-incidence condition, sharing and integrating regional expertise, multidisciplinary experience and defining consensus best-practice recommendations is particularly important. Well-coordinated collaborative efforts will ultimately pave the way to the development of evidence-based international guidelines.

## Background

Kennedy’s disease (KD), or X-linked Spinal and Bulbar Atrophy (SBMA), is a rare neurodegenerative disorder. The diagnostic possibility is typically raised when an adult man presents with slowly progressive lower motor neuron symptoms associated with signs of androgenic dysfunction such as gynecomastia [1–3]. The genetic cause was identified in 1991 as an abnormal expansion of CAG repeats in exon 1 on androgen receptor (AR) gene located on chromosome X at position q11–12 [1]. The objective of this paper is to present a national consensus guideline for the diagnosis and care of KD patients. The guideline has been developed under the auspices of the French ALS/MND Network (FILSLAN, Filière de Santé Maladie Rares pour la SLA et les Maladies du Motoneurone) and the French Health Authority (HAS, Haute Autorité de Santé) and coordinated by the first author. Our aim is the optimisation and harmonisation of diagnostic criteria, management pathways and monitoring protocols across France for KD. We present a systematic review of pharmacological treatments used at various centres, analyse key support needs, highlight gaps in service provision, and identify products and services which are currently not reimbursed.

## Results

### LITTERATURE review

#### Physiopathology

Kennedy’s disease is caused by CAG repeat expansions in the AR gene resulting in polyglutamine (polyQ) expansion in the AR protein. Expansions larger than 38 CAG trinucleotide repeats result in the formation of a pathological protein called polyQ-AR [3]. KD therefore belongs to the family of polyQ neurodegenerative conditions, which also includes Huntington’s disease, dentato-rubro-pallido-luysian atrophy (DRPLA) and six types of spinocerebellar ataxias [4]. It should be noted that unlike in Huntington’s disease, there is a mitotic andmeiotic stability of the CAG expansion in KD which explains the absence of anticipation phenomenon [5].

PolyQ diseases are characterised by the dysfunction and subsequent neuronal death of specific cell populations and the accumulation of toxic intracellular proteins [6]. The toxicity of polyQ-AR occurs through multiple cellular mechanisms: transcriptional dysregulation, mitochondrial dysfunction, disruption of protein homeostasis and cellular signalling pathways, as well as autophagy [7]. The AR, which is part of the nuclear receptor superfamily, is physiologically located in the cytoplasm and is bound to heat shock proteins. The binding of testosterone or dihydrotestosterone to its cytoplasmic receptor results in nuclear translocation and binding to androgen-responsive elements localised in the target gene promoter. Translocation of the polyQ-AR protein into the nucleus appears to be central to the pathogenesis of KD, because the deletion of the nuclear localisation signal limits toxicity in murine KD models [8]. KD is the only X-linked polyQ disease and only men develop the full spectrum of symptoms. Women with the mutation are typically asymptomatic, but minor manifestations such as fasciculations, cramps or subtle distal motor deficits are sometimes reported [9].

No disease-modifying therapy is currently available, but deciphering the pathophysiology cascade of KD allowed the development of a number of promising therapeutic strategies [1, 2, 10–13]. Interventions to reduce androgen levels have already been evaluated in humans [14, 15]. Other approaches, such as inhibition of AR gene transcription, focus on the preclinical stage of the condition [16]. Post-translational interventions, such as AR phosphorylation modulation and alteration of polyQ-AR protein toxicity, are also under development [17, 18].

#### Epidemiology

Kennedy disease is a low-incidence condition that is well documented worldwide. A haplotype study of 123 patients revealed that pathological CAG expansions the humans are not the result of a single founding event, but had emerged in several parts of the world independently [19]. Comprehensive epidemiological studies are lacking in KD. A detailed study of incidence and prevalence in the Veneto region of Northern Italy reported a prevalence of 2.58 / 100,000 (95% confidence interval 1.65–3.35) in the male population [20]. Based on 68 patients, five different haplotypes have been identified, confirming the presence of multiple founder effects. Some geographical regions have higher prevalence, such as the Vasa region in western Finland, where 13 per 85,000 male inhabitants are affected [19]. These figures probably underestimate the true prevalence of the disease because of the large number of un- or misdiagnosed patients worldwide. It has been shown that up to 2% of patients diagnosed as ALS actually have KD [21].

#### Natural history

Table 1 presents an overview of large descriptive series of KD. Natural history studies in KD [25, 28] are of paramount importance as they help to define screening protocols, plan multidisciplinary interventions and inform clinical trial designs. Symptom onset typically occurs in the mid-30s, but it varies significantly and cases as young as 4 [37] and as old as 78 [38] have been published in the literature. The spectrum of initial manifestations also varies considerably. Commonly reported initial symptoms include: cramps, fasciculations, tremor, dysarthria, dysphagia and gynecomastia. More rarely, the disease manifests in myalgia, unexplained fatigability, exercise intolerance, elevated creatine phosphokinase (CK), laryngospasm or genito-urinary endocrine syndromes, such as hypospadias, micropenis, or oligospermia [39]. The largest dataset on the natural history of KD is a well-documented series of 223 Japanese patients [24] which provides a general template of symptomatic milestones despite individual variations. Postural tremor of the upper limbs is a common early symptom which occurs around 33 years of age. Motor deficits then typically appear in the lower limbs at 44 years of age. Mobility is gradually affected and a ramp may be required to go up the stairs at around 49 years. Dysarthria is often experienced at around 50, followed by dysphagia at 54, walking aids are often required by 59 and many patients need a wheelchair by 61 [24]. The patient’s survival however is not significantly different from the background population: the 10-year survival was 82% versus 95% for controls, which was not statistically significant [25]. There are, however, extreme juvenile presentations with markedly rapid progression [40]. Patients often suffer from a circuitous diagnostic journey and considerable diagnostic delay. Diagnostic delay from symptom onset the confirmed diagnosis is in the order of 5 years based on a US series of 57 patients [26]. The implications of diagnostic delay are not sufficiently discussed in the literature, but misdiagnoses and unnecessary interventions, such as laminectomies and IvIg treatment are not uncommon [41].

**Table 1:** A list of large descriptive case series of KD in the literature (non-exhaustive list)

Author	Aim	Methodology, level of evidence	Population	evaluation parameters	Most significant results
Dejager et al., 2002 [22]	Description of endocrine and metabolic changes	Monocentric cohort study Level of evidence: IV	22 KD	Gynecomastia, blood hormonal, lipid and glucose assessment	Gynecomastia in 73% of cases, infertility or decrease of testicular volume in 60%, elevation of total cholesterol, LDL-C and triglycerides (54, 40 and 48%, respectively).
Sperfeld et al., 2005 [23]	Evaluation of the incidence of laryngospasm	Monocentric cohort study Level of evidence: IV	49 KD	Symptom questionnaire, respiratory tests	47% of the KD patients experienced laryngospasm.
Atsuta et al., 2006 [24]	Description of the natural history of KD	Multicentre cohort study Level of evidence: IV	223 KD	Clinical and biological parameters, Rankin score	Inverse correlation between the number of CAG repeats and the age of onset of symptoms
Chahin et al., 2008 [25]	Evaluation of functional decline and prognosis	Single centre case-control study Level of evidence: III	39 KD 70 Controls	10-year survival rate and functional status (ALSFRS-R) at last follow-up	Survival rate of KD was not significantly altered compared with controls (82% vs 95%, *p* = 0.053). The functional status was relatively preserved. Patients are mostly limited for climbing the stairs. Bulbar symptoms in all patients but no need for gastrostomy. Non-invasive ventilation was needed in one single patient.
Rhodes et al., 2009 [26]	Description of the natural history of KD	Single centre cohort study, patients participating in the Dudasteride therapeutic trial. Level of evidence: IV	57 KD	Neurophysiological, biological, neuropsychological and quality of life parameters	Long diagnostic delay (5 years). Correlation between androgen levels and muscle strength.
Soukup et al., 2009 [27]	Evaluation of cognition changes in KD	Monocentric case-control study Level of evidence: III	20 KD 20 Controls	Neuropsychological assessment evaluating executive functions, memory, attention	Existence of a subclinical impairment of frontal and temporal functions
Hashizume [28]	Characterisation of the natural history of KD	Monocentric cohort study Level of evidence: IV	34 KD	Quantitative outcome measures including functional and blood parameters	Disease progression is not affected by CAG repeat length Objective motor functional tests such as the 6-min walk test and grip power or serum creatinine levels are more sensitive at an early stage than by the functional rating scales
Araki et al., 2014 [29]	Evaluation of ECG abnormalities	Monocentric cohort study Level of evidence: IV	144 KD	ECG parameters	ECG abnormalities in 49% of cases, mainly consisting in ST segment anomalies in V1-V3 (19%) and V5-V6 (18%). Brugada syndrome (12%) with two cases of sudden death
Querin et al., 2015 [30]	Characterisation of the extraneurological profile of KD	Multicentre cohort study Level of evidence: IV	73 KD	Biology, androgen sensitivity index, genito-urinary symptoms, dual-energy X-ray absorptiometry, muscle biopsy	Androgen insensitivity. Increased prevalence of genito-urinary symptoms and diminution of bone mass.
Bertolin et al., 2016 [31]	Genotype-phenotype associations	Multicentre cohort study Level of evidence: IV	159 KD	Correlation between the number of CAG repeats and motor function	No genotype/phenotype correlations
Nordenvall et al., 2016 [32]	Establishing the incidence of hypospadias	Data analysis from a national KD registry Level of evidence: IV	4 KD	Association between hypospadia and KD	Hypospadia in KD may be underestimated
Francini-Pesenti, 2018 [33]	Evaluating the prevalence of metabolic syndrome	Monocentric cohort study Level of evidence: IV	47 KD	Metabolic syndrome Insuline resistance Non-alcoholic liver disease	High prevalence of insulin resistance, metabolic syndrome and non-alcoholic liver disease and NAFLD in SBMA patients
Rosenbohm et al., 2018 [34]	Evaluating the prevalence of metabolic changes	Monocentric cohort study Level of evidence: IV	80 KD	Panel of 28 laboratory parameters	Diabetes, hyperlipidemia and androgen insensitivity
Marcato et al., 2018 [35]	Establishing the prevalence of cognitive changes	Monocentric cohort study Level of evidence: IV	64 KD	Battery of neuropsychological test	Absence of neuropsychological abnormalities
Spinelli et al., 2019 [36]	Characterising cerebral radiological alterations	Monocentric case-control study. Level of evidence: III	25 KD 24 Healthy 25 ALS 35 Lower motor neuron-predominant conditions	MRI parameters: cortical thickness and diffusion tensor imaging (DTI)	Absence of abnormalities of the cerebral gray and white matters in KD patients.

*Abbreviation: ECG* electrocardiogram, *ENMG* electroneuromyogram, *MRI* magnetic resonance imaging

#### Genotype-phenotype correlations

An inverse correlation exists between CAG repeat numbers and age of symptom onset [24]. If non-motor manifestations are considered, this association is not evident [39]. The correlation between CAG repeat numbers and motor disability may only account for 60% of the observed clinical heterogeneity indicating that other factors; genetic, epigenetic or environmental, may play important roles in the development of the clinical syndrome. This is illustrated by the presence of considerable phenotypic variability within the same family despite identical CAG repeat numbers [3]. Correlation analyses between CAG repeat numbers and electrophysiological indices have been inconsistent. High repeat numbers are thought to be predominantly associated with motor abnormalities, whereas low repeat numbers primarily manifest in sensory changes [42]. In a recent study, CAG repeat lengths were negatively correlated with motor amplitudes, but not with sensory amplitudes [43]. Finally, in a cohort of 155 Chinese KD patients both motor and sensory electrophysiology indices correlated with repeat lengths [44].

##### Animal models

A number of transgenic animal models have been successfully developed to aid KD research. The AR-65 model, carrying 65 CAG repeats showed a mild phenotype compared to the AR-120 (120 repeats) which exhibited both motor and behavioural symptoms. The motor symptoms observed in AR-120 were associated with spinal alpha-motor neuron degeneration, without evidence of cerebral changes [45]. In another transgenic mouse model, overexpression of wildtype AR exclusively in skeletal muscle fibres without polyQ expansions resulted in neuromuscular atrophy, with altered muscle morphology, androgen-dependent muscle weakness and early death [46]. This model highlights that muscle-based therapeutic strategies may be effective in KD. A landmark animal-model study showed that the manipulation of the nuclear localization signal of polyglutamine-expanded AR improves muscle function and demonstrated that pharmacologically induced autophagy may rescue motor neurons from mutant AR toxicity [8].

#### Diagnosis, neurological and non-neurological manifestations

##### Neurological symptoms and signs

Motor and sensory manifestations

Muscle weakness is a key clinical feature of the disease; it is present in 97% of cases and usually manifest around the age of 35–40. Higher repeat numbers are associated with younger age of onset, but no association has been found between repeat numbers and rate of progression [24, 47, 48]. The very first symptom typically experienced is a postural hand tremor, which is usually mild and appears around the age of 33 [24]. Head and voice tremors [48, 49] and lower limb tremors may also be observed [50]. Tremor is often alcohol-responsive [51] and is modulated by load-bearing and posture [52]. Motor deficits initially present in the lower limbs in 70% of cases, in the upper limbs in 31%, in the bulbar region in 11% and facially in 2% of cases, but symptoms can also present in multiple body regions simultaneously [24]. In the lower limbs, the motor deficit is usually proximal, bilateral and symmetrical and is accompanied by decreased or absent deep-tendon reflexes. Distal weakness however has also been reported early in the course of the disease [53]. Cramps, myalgia and fasciculations are particularly common [48, 54]. KD is also associated with widespread sensory involvement [55] which often results in decreased perception of vibration [56]. Fatigability is often noted on initial assessment [48] which may be fluctuating and the possibility of myasthenia gravis as a differential diagnosis is sometimes raised, especially since repetitive stimulation may be abnormal and improvement after anticholinesterase inhibitor therapy has been reported [57]. Bulbar symptoms typically follow limb manifestations and start with dysarthria or dysphonia before dysphagia ensues [24]. Cases of pure bulbar onset however have also been reported [58].

Lingual fasciculations are a common feature of KD [49, 54] and perioral and chin fasciculations may appear as myokymia [59]. These may be visible at rest, but tend to become obvious when the patient is asked to whistle or protrude the lips [49]. Lingual atrophy only occurs later in the course of the disease, but lingual mobility tends to be relatively preserved [59]. The tongue may develop an unusual shape due to coexisting denervation and reinnervation. The striking contrast between relatively mild bulbar symptoms (dysarthria and dysphagia) and the considerable lingual atrophy and fasciculations is a hallmark clinical sign [49]. Clinically there is often no overt impairment in heat or pain sensation, despite evidence of small sensory fibre degeneration histologically [60]. Laryngospasm may occur with a sense of choking. Patients feel that the air cannot enter or exit the airways for long seconds. This often followed by stridor secondary to the rapid and vigorous contraction of the laryngeal sphincters. This is a common (up to 50% of the patients), frightening and hugely distressing symptom, but rarely escalates to prolonged or serious episodes. When laryngospasms are reported, a thorough workup for gastroesophageal reflux disease is recommended [48]. Sleep disorders and obstructive sleep apnoea are also commonly experienced, but the exact aetiology, neurological and respiratory mechanisms have not been fully elucidated to date [61]. Subclinical oculomotor dysfunction may occur, which typically remains asymptomatic and can only be detected on targeted examination [62].

Neuropsychological abnormalities

The link between cognitive deficits and KD is not well established despite sporadic reports [63–65], and the fortuitous associations cannot be excluded. Nevertheless, the presence of mutated AR protein has been shown in non-motor brain regions [66]. Furthermore, specific psychological traits such as lack of self-confidence, emotional flattening and poor concentration is often observed [27]. Some neuropsychological studies captured executive impairment with short and long-term memory deficits [27, 67]. These abnormalities however generally remain subclinical and do not significantly interfere with the daily conduct of the patient. A more recent study highlighted deficits in social cognition, impaired empathy on theory of mind tests [68]. Interestingly, a larger study including 64 KD patients did not detect any abnormalities on detailed neuropsychological examination [35]. It is noteworthy however that most neuropsychology studies focus on cognitive testing, particularly on executive function and compared to other motor neuron diseases [69, 70], detailed behavioural assessment is seldom performed.

##### Extra-neurological involvement

Endocrine changes

Endocrine involvement stems from partial androgen insensitivity and often precedes neurological manifestations. Gynecomastia may be present in up to 73–78% of male KD patients [22, 30]. It typically becomes apparent after puberty [22], and is cause by a decreased response to androgen which, under physiological circumstances, exerts an apoptotic effect on the breast tissue. Erectile dysfunction and decreased libido are also common, reported in 40–50% of cases [22, 26]. Frank exocrine testicular involvement, i.e. infertility and testicular atrophy can be found in around 60% of patients [22]. One of the most disease-specific endocrine indices in KD is the androgen sensitivity index (ASI), which is elevated in 64% of cases [22]. ASI is calculated as; ASI = LH (mIU/ml) x Testosterone (ng/ml). Baseline testosterone levels tend to be variable, and reports are inconsistent. Increased, normal and low baseline testosterone levels were all reported. Baseline levels of LH and FSH are typically high or normal, and only seldom decreased. Using the GnRH dynamic test, the luteinizing hormone (LH) response may be dramatic (more than 5 times the baseline) in 36% of cases [22]. These findings indicate both endocrine and exocrine testicular dysfunction. Increased LH coupled with normal or high testosterone levels specifically indicates partial androgen resistance as physiologically, androgens downregulate LH secretion. Elevated estradiol levels were reported in 39% of KD patients in one study [26], but this was only detected in 9% of cases in another series [22]. Finally, an association was suggested between hypospadias and KD but this has not been firmly established [32, 71, 72].

Metabolic alterations

In a large Italian series, 41% of patients had abnormally high fasting serum glucose levels [30], but this has not been confirmed by other studies [22, 26]. Most studies support an increased incidence of hyperlipidaemia compared to the background population [22, 26, 30, 34]. An increased risk of insulin resistance, metabolic syndrome and non-alcoholic liver disease has been suggested recently [33]. The safety and efficiency of statin therapy has not been systematically studied in KD. According to the French Health Authority, there is not a blanket contraindication to prescribe statins in all neuromuscular conditions, but in certain genetic muscle diseases, autoimmune myopathies (Statin-induced necrotizing autoimmune myopathy -SINAM) or in patients with a prior adverse reaction to a cholesterol-lowering agent, the careful evaluation of risk / benefit ratio and attentive monitoring is required. [73]

Bone involvement

In a KD series, 5% of patients had femoral osteoporosis, 36% lumbar and/or femoral osteopenia measured by bone osteodensitometry (59). Compared to healthy subjects, mean bone mineral density (BMD) in KD patients was lower in the femur and preserved in the lumbar vertebrae. Decrease BMD may be secondary to relative inactivity due to lower limb weakness. However, there is a lack of correlation between motor disability and lumbar BMD. It is also noteworthy that up to 65% of KD patients have vitamin D deficiency. The role of partial androgen resistance remains to be explored.

Cardiac involvement

Potentially life-threatening conduction abnormalities have been repeatedly reported [29, 30] manifesting in Brugada-type ECG alterations with ST elevation in the precordial V1 to V3 leads, forming a characteristic ‘coved’, upward convex ST pattern. This may be associated with the severe ventricular tachyarrhythmias potentially predisposing to sudden cardiac death. In one of the largest series of 144 KD patients, the prevalence of Brugada-type ECG abnormalities was 4% and linked to two cases of sudden cardiac death [29]. Underlying molecular channelopathies have been proposed, such as the abnormal expression of the cardiac sodium channel Na(v)1.5, linking KD to Brugada-type conduction abnormalities. Despite the scarcity of reports, these data warrant careful screening measures and preventive management. Subclinical dysautonomic manifestations may also be possible [74]. However, orthostatic hypotension has only been identified in patients with very high CAG repeat numbers (68 CAG repeats) [75]. It should be specifically noted that there are no reports of cardiomyopathy in KD [76].

Genito-urinary disorders

A large study identified a considerable frequency of genitourinary disorders among 73 KD patients using the International Prostate Symptom Score (IPSS) questionnaire [30]. Moderate dysuria was detected in 30% of patients, while severe dysuria was reported in 10% of cases. Moreover, three patients required a suprapubic catheter. The exact mechanism remains unknown, but a number of hypotheses were put forward such as androgen insensitivity may unmask bladder obstruction [77], direct detrusor and perineal muscle dysfunction, and dysautonomia was also implicated. As noted earlier, erectile dysfunction is particularly common in KD, but few prospective studies evaluated its prevalence.

##### Laboratory examinations

Electrophysiology

The electrophysiological features of KD encompass diffuse, chronic neurogenic changes and fasciculations beyond the regions overtly affected on clinical assessment [59]. An important cue suggestive of KD is the presence of decreased sensory amplitudes that is present in 72 to 100% of cases [26, 48, 49, 54]. Electrophysiology is also useful in the differential diagnosis of the disease, in particular to distinguish KD from ALS in which sensory abnormalities are typically absent [78]. EMG abnormalities are usually upper limb predominant [42]. The presence of decrement on repetitive stimulation is not unusual, and may be mistaken for myasthenia gravis [79]. Activity-dependent conduction blocks on single fibres have been linked to muscle fatigability [80]. Anecdotally, the presence of giant F waves seems to be more common in KD than in ALS [81]. Clinical trial designs favour motor unit number estimation (MUNE) [82] and the “cluttering index” [83] which reliably capture the gradual decline in functioning motoneurons.

Muscle biopsy

Muscle biopsy is not commonly performed and is seldom necessary to establish the diagnosis. It typically reveals both myopathic (nuclear centralisation, myofibrillar disorganisation) and neurogenic (angular fibres or type grouping) changes [84]. These abnormalities may occasionally be useful for differentiating KD from diagnosis with ALS [85].

Serum markers

Increased creatine kinase (CK) levels may be detected in 80 to 94% of KD patients, often in association with increased transaminases (24). CK values range from 31 to 4955 IU / L, with an average serum level of 863 IU / L (normal < 200 IU / L) [24]. Rarely, the work-up for an unexplained elevation of CK and transaminase level may lead to the diagnosis of KD [86]. Decreased serum creatinine is thought to strongly correlate with clinical disability [87] and may already be detected in the preclinical phase of the disease [88]. Other serum markers are primarily endocrine indicators which are separately discussed in paragraph 5.2.1.

Imaging

Few dedicated imaging studies have been performed to investigate cerebral alterations in KD [89]. Imaging studies focus on cerebral alterations [90, 91], which is surprising given the lower motor neuron predominant involvement in KD. Qualitative [92] and quantitative [93, 94] spinal imaging are increasingly used in other motor neuron disease to characterise spinal grey and white matter alterations [95], but these techniques have not been applied to KD cohorts to date. Existing cerebral studies have primarily used tractography and morphometry and identified widespread white matter and grey matter abnormalities compared to controls [90, 91, 96]. In addition to the existing brain studies, innovative studies also examined the biomarker potential of muscle MRI [97, 98]. Despite these efforts, no unifying KD imaging signature has been established to date, and a recent imaging study failed to capture differences between patients and healthy controls [36]. Given the sexual dimorphism of neuroanatomy [99–101], KD imaging studies should ideally include male controls alone. It is also noteworthy that unlike the longitudinal studies published in other motor neuron diseases [102], KD imaging studies are cross-sectional and patients are included in different phases of their disease [103]. The inclusion of clinically heterogeneous cohorts precludes the identification of unifying imaging signatures [104] and the systematic characterisation of extra-motor involvement [105]. Most imaging studies are structural and prospective functional and connectivity studies are lacking [106].. While previous studies of lower motor neuron predominant motor neuron diseases suggested cortical compensation [107] or a degree of functional re-organisation in response to structural degeneration [108], no such studies have been undertaken in KD [109]. A positron emission tomography (PET) study identified regional hypometabolism of in the frontal lobes without associated neuronal loss [110]. An innovative study demonstrated that muscle MRI differentiates KD and ALS, and muscular MRI alterations correlate with disease severity, suggesting that muscle MRI may have a putative biomarker role in KD [111]. Despite clinical descriptions of mild symptoms, imaging studies of female homozygous CAG expansion carriers are lacking [112]. Missed opportunities imaging include the lack of longitudinal [113], presymptomatic [103], prognostic [114], machine-learning [114–117], striatal [118], brainstem [119], spinal cord [120, 121], and region of interest [122] studies, all of which have been successfully conducted in other motor neuron diseases and evaluated from a biomarker perspective.

#### Differential diagnosis

There are rare reports of bulbar and spinal amyotrophies without CAG triplet expansions in AR gene. An autosomal dominant non-X-linked form has been reported with spinal and bulbar involvement with associated gynecomastia [123]. In clinical practice however, the main differential diagnosis of KD is ALS, which can be readily distinguished from KD due to the more rapid functional decline, the presence of upper motor neuron signs, the absence of sensory neuropathy and endocrine disorders (Table 2). Less likely differential diagnoses include conditions with lower motor neuron presentations, such as Hirayama disease [124], multifocal motor neuropathy, spinal muscular atrophy (SMA) [125], post-polio syndrome (PPS) [126], CIDP, progressive bulbar palsy (PBP) [41], progressive muscular atrophy (PMA), spinal cord ischaemia [92], myelopathies [127] and Charcot-Marie-Tooth disease, but most of these conditions have specific radiological, molecular or electrophysiological features which help to distinguish them from KD. The other differential diagnoses depend on the initial constellation of clinical signs or symptoms which are often extra-neurological (Table 3).

**Table 2:** Differential diagnosis between ALS and KD. The interpretation of these simple criteria must be put in perspective with the phenotypic heterogeneity of these two pathologies and more particularly of ALS (adapted from Pradat, 2014)

Sex and age	KD	ALS
	Adult man in his thirties or forties	Adult man or woman in their fifties or sixties
Genetic	X-linked transmission Family history found in 2/3 of the cases	Familial in 10% of cases (autosomal dominant, recessive, multigenic, exceptionally X-linked)
**Neurologic signs**		
**Limb involvement**		
Topography Predominance in LL vs UL Proximal vs distal	Symmetrical LL more affected Proximal predominance	Most often asymmetrical LL = UL Proximal = distal
Pyramidal syndrome	Absent	Present in most patients
**Bulbar involvement**		
Fasciculations	Lingual quasi constant lips, chin, or perioral area (often with a myokymic appearance).	Lingual frequent
Lingual atrophy	Present with a reshaped aspect	Present
Dysarthria and dysphagia	Moderate, contrasting with the severity of lingual atrophy and slowly progressive	Evolutive (frequent use of assistive devices for communication and enteral nutrition)
Pseudo-bulbar involvement	absent	possible
Cognitive involvement	Absent or very slight	Association with fronto-temporal dementia in 5–10% of the cases.
Respiratory insufficiency	Rare	Common
Progression	Slow Normal life expectancy	Rapid (but slow progressive forms) Median survival of 3 years
Gynecomastia	Frequent	Absente
ENMG	Chronic motor denervation associated with a decrease of sensory potentials	Motor denervation with frequent signs of activity (fibrillation) and normal sensory potentials
CPK	Important elevation	Elevation

*Abbreviations: CPK* creatine phosphokinase enzyme, *ENMG* electroneuromyogram, *LL* lower limbs, *UL* upper limbs

**Table 3:** Differential diagnosis of KD depending on the presentation and predominant signs and symptoms

Clinical features	Differential diagnosis
Isolated damage to the peripheral motor neuron	- Other causes of spinal amyotrophy (particularly related to SMN1 mutation), - Spinal motor forms of Charcot-Marie-Tooth neuropathy - Progressive muscular atrophy - Post-polio syndrome
Cramps and fasciculations	-"Benign” cramps-fasciculations syndrome - Isaacs syndrome
Progressive bulbar involvement:	- Myasthenia gravis - Congenital myopathies - Oculopharyngeal muscular dystrophy;
Proximal motor deficit associated with a rise in CPK	- Progressive muscular dystrophies - Congenital myopathies - Muscular canalopathies - Inflammatory or metabolic myopathies;
Amyotrophic motor deficit associated with sensory impairment	Charcot-Marie-Tooth neuropathy

#### Management

In the absence of effective disease-modifying therapies, the mainstay of management is symptomatic support; pain management, physiotherapy, speech therapy [128], gastrostomy etc. The benefits of multidisciplinary management have not been systematically evaluated in clinical trials, but empirical evidence suggests considerable benefits. No best practice recommendations can be identified for KD at national, European or international level.

The identification of the genetic underpinnings of KD has paved the way for robust in vitro studies, then to the development of in vivo models [129–131] which subsequently led to a series of promising pharmaceutical trials in humans [13]. Animal studies identified androgen reduction as candidate therapeutic strategy. The first hormonal therapy evaluated in humans was leuprorelin, which reduces testicular testosterone production by lowering gonadotropin levels [15, 132]. A study showed no benefit on muscle function parameters but was suggestive of improved swallowing function. Another drug that has been evaluated is dutasteride, a selective 5 alpha-reductase inhibitor, used in the treatment of prostate adenomas, which blocks the enzymatic conversion of testosterone to dihydrotestosterone, but it showed no benefit [14]. Other treatment strategies aimed at improving muscle function directly either through exercise, or through anabolic drugs, such as clenbuterol [133, 134], with disappointing outcomes. A recent study evaluated the safety, tolerability, and preliminary efficacy of an IGF-1 mimetic, but treatment was associated with a high incidence of immunogenicity without clear improvement in muscle function [135].

### Consensus statement on the diagnosis and care of KD patients

#### Diagnosis and clinical evaluation

##### Members of the multidisciplinary medical team

The general practitioner has a central role to identify the initial symptoms of KD and promptly refer the patient to a neurologist to initiate the appropriate investigations and to confirm the diagnosis. The initial evaluation is typically performed in a neurology department, preferably at a tertiary referral centre for motor neuron diseases, with the assistance of other medical specialists; endocrinologists, geneticists, ENT specialists, pulmonologist, cardiologists, dieticians, and rehabilitation specialists. A number of other health care professionals are also indispensable for the evaluation and supportive management of KD, such as clinical psychologists, neuropsychologists, physiotherapists, occupational therapists, dieticians, speech therapists and social workers.

##### Circumstances supporting the suspicion of the diagnosis

A family history suggestive of X-related inheritance should be sought but may not always be present.

##### Neurological signs and symptoms

The commonest presentation is the constellation of progressive motor deficits in an adult man around the age of 40, typically affecting the lower limbs proximally, with fatigability when walking, difficulty climbing the stairs, in conjunction with cramps and fasciculations. Postural tremor of the upper limbs can sometimes precede the motor manifestations. Signs of bulbar involvement, dysarthria, dysphonia, and dysphagia and/or laryngospasm usually present later in the course of the disease but would be suggestive of the diagnosis if they are present in combination with limb weakness. The diagnosis should be suspected based on the constellation of a range symptoms and neurological findings which are presented in Table 4.

**Table 4:** Neurological signs suggestive of Kennedy’s disease

Site of involvement	Neurological signs
Peripheral motor neuron involvement limited or diffused to the four limbs	- Fasciculations - Cramps - Amyotrophy - Predominant motor deficit in the lower and proximal limbs, - Decrease or abolition of osteo-tendinous reflexes
Progressive peripheral bulbar involvement	- Atrophy of the tongue and fasciculations that may extend to the lips, chin, or perioral area (often with a myokymic appearance). - Dysarthria, with potentially a discreet nasal voice - Dysphagia
Sensory involvement	- Most often subclinical and distal
Absence of evidence of central motor neuron involvement	- Absence of spasticity, clonus, exaggeration and / or diffusion of reflexes, or Babinski’s sign
Postural tremor of the upper limbs	

##### Extra-neurological signs and symptoms

Gynecomastia, which is usually bilateral and symmetrical, is a common early clinical cue which is supportive of a suspected diagnosis. Other signs of hypogonadism, such as erectile dysfunction or infertility, may also constitute initial symptoms, but they typically only raise the suspicion of KD if they are associated with frank neurological deficits. Abnormally high serum creatine kinase (CK) is seldom indicative as it may be caused by a plethora of other medical conditions. CK values of 2–4 times the normal level can be observed, but this is not specific to KD. Similarly, a moderate raise in transaminases (ASAT, ALAT) is often observed but not specific.

##### The confirmation of the diagnosis

Electrophysiology

This is an essential part of the diagnostic work-up and electrophysiological findings need to be carefully interpreted in the context of clinical findings. Diffuse and chronic neurogenic changes are found with widespread fasciculations. Electrophysiological abnormalities typically extend well beyond the region affected clinically, unravelling extensive subclinical involvement. An electrodiagnostic cue is the decrease of sensory amplitudes (sensory neuronopathy) in a non-length-dependent pattern in association with the above motor anomalies.

Genetic testing

KD is caused by an abnormal CAG triplet expansion in exon 1 of the AR gene on the X chromosome. The diagnosis of KD is established above a repeat number of 38. In suspected cases, a blood sample should be sent to a molecular genetics laboratory, accompanied by a request document, a family tree, a consent form signed by the patient and a signed consultation request. It is preferable that genetic testing takes place in certified laboratory. The result must be communicated by a qualified practitioner during a formal consultation allowing plenty of time for questions and offering the opportunity to ask further questions on follow-up. The risk of passing on the mutation to male members of the family must be discussed with clarity. Specific local guidelines may exist on how to communicate genetic results to those affected and a signature may be requested from the patient to acknowledge that the results have been communicated. Institutional and national legal and ethical frameworks may govern local genetic counselling procedures.

Differential diagnoses

The main differential diagnosis of KD is amyotrophic lateral sclerosis (ALS), which carries a worse prognostic outlook than KD. ALS differs from KD in a much faster evolution, the presence of upper motor signs, the absence of sensory neuropathy and the lack of endocrine manifestations (Table 4). Other differential diagnoses depend on initial presentations, such as endocrine features (Table 5).

**Table 5:** Levels of evidence according to the French Health Authority guidelines

Level of evidence	Criteria
Level 1	Comparative and randomised studies with appropriate statistical power Meta-analyses of randomised studies
Level 2	Comparative and randomised studies with limited statistical power Non-randomised studies with high methodological value Cohort studies
Level 3	Case-controls studies
Level 4	Comparative studies with known bias (selection, inclusion, convenience sampling) Retrospective studies

##### The announcement of the diagnosis and patient information

Breaking the news of the diagnosis should take place in a quite institutional environment with no distractions by an experienced physician who can dedicate sufficient time to answer any questions about the implications of the diagnosis will patience, empathy and humanity. Following the consultation, it is paramount that the patient’s general practitioner is sent a formal report. It is also essential that a follow-up appointment is organised to allow the patient and the caregivers to ask further questions and to evaluate the patient’s understanding of the diagnosis. The discussion of the foreseeable disability, projected rate of progression, management options and lack of effective pharmaceutical interventions should be balanced, factual and frank. Beyond discussing therapeutic options, initial consultations should set realistic goals, put a long-term management plan into place to allow efficient adaptation to the disease. Instead of merely conveying factual medical information, active listening, offering support, getting to know personal, social and professional circumstances are vital to establish trust and develop individualised adaptation strategies.

##### The systematic evaluation of disability, comorbidities, and prognostic considerations

Evaluation of neurological signs

A focused neurological examination informs the choice of mobility aids, occupational therapy interventions and helps to enlist the help of the appropriate allied health care professionals. Proximal lower limb weakness is a cardinal symptom in the vast majority of cases leading to difficulty getting up from a sitting position, climbing the stairs and overall poor mobility. Muscle strength testing alone underestimates the functional disability experienced by the patient and it also overlooks the fatigability associated with the condition. Dysphagia impedes the oro-pharyngeal processing of the alimentary bolus and may result in apprehension of eating and adverse nutritional consequences. Choking episodes are a rare occurrence, but dysphagia can lead to recurrent aspiration pneumonias nonetheless. Dysarthria typically remains relatively mild and seldom leads to the loss of oral communication and reliance on augmentative and alternative communication aids. A number of disease-specific and generic instruments have been validated to assess limb and bulbar disability [2, 113, 128]. The most commonly used assessment scale is the SBMA functional rating scale (SBMA-FRS) [136] but manual muscular testing scores (MRC) are also routinely documented (Supplementary material 2). Cognitive impairment has been reported in KD, especially attention deficits and dysexecutive manifestations. These are typically mild and may only be detected on target neuropsychological examination. Given the lower motor neuron predominance of the condition pseudobulbar affect is not a feature of KD [137, 138].

Respiratory and nutritional evaluation

Respiratory involvement is often subclinical and may manifest either as an inspiratory weakness or weak cough due to additional abdominal wall muscle involvement. Respiratory insufficiency may become apparent after lower respiratory tract infection or following a surgery. It can be complicated by acute respiratory insufficiency, requiring hospitalisation, intensive care or transient respiratory support. Specialist respiratory advice should be sought in cases of orthopnoea, evident diaphragmatic involvement, productive cough, coexisting chronic obstructive pulmonary disease, and recurrent upper respiratory tract infections. The initial respiratory assessment should include formal spirometry in both sitting and lying positions with the measurements of maximum respiratory pressures (PImax and PEmax) and a sniff nasal inspiratory pressures (SNIP). Cough weakness may be suspected based on reduced inspiratory and expiratory pressures, but must be confirmed by peak cough flow measurements. Early morning arterial blood gas analysis and overnight night oximetry are also part of the respiratory work-up.

Nutritional evaluation

Formal nutritional assessment should begin with the recording of anthropometric data; weight, height, body mass index (BMI); tissue body composition (fat mass and lean mass) using validated scales and instruments such as the bioimpedancemeter. Standardised food preference and dietary surveys also help to discuss diet adjustments, increase caloric intake and choose food supplements.

Endocrine and metabolic evaluation

Gynecomastia is evident on clinical examination. If it is unilateral, ultrasound or mammogram should be considered to exclude an underlying neoplastic process, If there is a clinical suggestion of hypogonadism based on the clinical history (decreased libido, erectile dysfunction) or evidence of testicular atrophy or gynecomastia a typical hormone panel would should include: total testosterone level, estradiol level, luteinizing hormone (LH) and follicle stimulating hormone (FSH) levels at baseline, and gonadotrophin-releasing hormone (GnRH). Unlike in other motor neuron diseases [139], fecundity in KD is under evaluated. In case of infertility, referral to a reproductive medicine specialist should be made and further tests such as spermogram may be required. The initial metabolic screen should include fasting glucose, HbA1c, LDL, HDL, total cholesterol, triglycerides, and transaminases. Depending on these abnormalities, the assessment of cardiovascular risk will be carried out.

Cardiac evaluation

A 12-lead ECG should be recorded on each newly diagnosed KD patients as a first line screening for repolarisation abnormalities, such as the Brugada pattern, which is characterised by coved-type ST elevation from V1 to V3 leads. This ECG pattern may be indicative of increased risk for potentially life-threatening ventricular arrhythmias. ECG should ideally be reviewed by a cardiologist and additional tests such as Holter-monitoring, even monitors, loop recorders, echocardiograms may be required.

Bone assessments

As bone mineralisation abnormalities have been repeatedly reported in KD, a baseline bone densitometry scan (DEXA) and serum tests for 25-OH vitamin D, calcium and phosphate are also recommended.

##### Genitourinary disorders

Erectile dysfunction is not the only genitourinary symptom of KD, and additional urodynamic evaluation may be needed obstructive symptoms are reported.

##### Genetic counselling

A formal genetic counselling session will specifically discuss the risk of family members developing KD and the projected age of onset in those at risk. The X-linked recessive mode of transmission should be explained with clarity and genetic screening should be offered to family members at risk. Those at risk will be informed that only a CAG repeat number above 38 is likely to lead to the clinical spectrum of KD. The clinical manifestations of a repeat number between 35 and 37 are difficult to predict, but the clinical profile of affected family members may serve as a pointer. Genetic counselling will also inform couples at risk regarding the probability of passing on this genetic variant to their children. During counselling it is explained that there is no direct father to son transmission and that sons of female carriers are at 50% risk to inherit the genetic abnormality. Implications for prenatal or pre-implantation diagnosis are also explained during genetic counselling.

#### Management

##### General objectives

In the absence of effective disease-modifying treatments in KD, management centres on supportive interventions for the spectrum of neurological, respiratory, nutritional, endocrine, metabolic or cardiac symptoms associated with KD. The overarching objective of any intervention is to maintain independence, autonomy and dignity irrespective of physical disability while respecting individual care preferences. Key management strategies include:

- Passive or active mobilisation physiotherapy to prevent the painful musculoskeletal complications of poor mobility.

- Preserving autonomy by helping patients to adapt to physical disability through individualised occupational therapy (home modifications, adapted kitchen utensils, taps, keyboards) and mobility aids, such as wheelchairs, sticks, walking aids, orthoses, stair lifts etc.

- Individualised speech therapy to preserve oral communication in patients with dysarthria.

- Teaching compensation strategies for swallowing disorders, such as adopting a safety posture, slow swallow, small boluses intake, avoidance of talking while eating etc.

- Monitoring nutritional status, dietary modification to suit individual bulbar performance, initiation of food supplementation, high calorie supplements and vitamins, adjusting food textures (avoidance of dry crumbly foods), changing food consistency with thinkers if needed (fluids). Considering gastrostomy placement in sever dysphagia (rarely needed).

- Monitoring respiratory function and anticipation of respiratory tract infections, initiation of cough-assist, breath stacking, non-invasive ventilation if needed.

- Management of extra-neurological complications, endocrine, metabolic and cardiac disorders; androgen resistance, hypogonadism, diabetes, dyslipidaemia, arrhythmias etc.

- Offering education sessions to patients and caregivers by social workers re: genetic risks, prognosis, financial advice, car adaptations, government grants, home modifications, equal opportunity employers.

- Offering psychological support to patients and caregivers re: adaptation, coping, and outlook.

##### The members of the multidisciplinary team

The management should ideally be coordinated by a neurologist at a centre specialised in motor neuron diseases and a general practitioner in the community. The neurologist confirms the diagnosis, breaks the news and coordinates the care throughout the course of the disease. The management however is strictly multidisciplinary and involves geneticists, endocrinologists, respiratory physicians, dieticians, ENTs, cardiologists, and rehabilitation physicians. The general practitioner ensures that the patient is linked in to a specialist centre, maintains a dynamic partnership with the neurologist and manages intercurrent symptoms, such as infections, diabetes etc. Hospital and community-based allied healthcare professionals, such as physiotherapists, occupational therapists, speech therapists, dieticians and social workers perform regular evaluations, and help the patient to adapt to disability to maintain independence and autonomy. Psychological support by an experienced clinical psychologist should be offered at the time of diagnosis and offered throughout the course of the disease. Specialist nurses affiliated with motor neuron disease centres also often offer invaluable advice and support to patients and caregivers and can assist with medical and administrative challenges. Patient organisations and charities offer additional support in the patient’s community or at a national level, by providing educational sessions, telephone helplines, websites, maintaining a network where patients and caregivers can interact and share information. Unlike in other motor neuron diseases [140], specialist palliative care interventions are seldom required [141] and patients don’t typically avail of hospice services. Specialist palliative care physicians and pain specialists can however offer consultations regarding management of respiratory insufficiency and musculoskeletal pain.

##### Therapeutic interventions

Before prescribing of any drugs, the increased risk of cardiac repolarisation abnormalities should be carefully considered. If there already is ECG evidence of an underlying repolarisation abnormality, a cardiologist should be consulted about the potential proarrhythmic effect of the new drug.

The pharmacological management of pain

Muscle cramps cause significant discomfort and quinine derivatives and magnesium are commonly prescribed. Even in the absence of established repolarisation disorders, mexiletine, a sodium channel blocker, should only be considered after consultation with cardiology should only be considered after consultation with a cardiologist regarding risk/benefit ration and to put careful monitoring in place. Pain due immobility and skin breakdown at pressure sites are other common cause of pain and the choice of analgesics should follow local guidelines and depend on tolerability of the side-effect profile of specific medications. Paracetamol, nonsteroidal anti-inflammatory drugs (NSAIDs), gabapentin, pregabalin, tramadol, selective serotonin reuptake inhibitors (SSRIs), serotonin–norepinephrine reuptake inhibitors (SNRIs), tricyclic antidepressants (TCA) are just some of the pain modulators used in KD, but the choice of specific drugs has to be guided by comorbidities (renal impairment, risk of dependency) and life style factors (driving, operating machinery). Any pharmacological treatment must be initiated after, or used in conjunction with non-pharmacological interventions, such as physiotherapy, mobilisation, massage, and occupational therapy. Alternative non-pharmacological interventions such as acupuncture may also be trialled depending on individual patient preferences.

Treatment of fatigue and mood disorders

Fatigue is a very common symptom, and is likely to be multifactorial due to muscle weakness, sedating medications, subclinical respiratory weakness, poor sleep or poor caloric intake. Accordingly, treatment will depend on the key aetiology, and the targeted correction of underlying factors may ameliorate fatigue. Modafinil or levocarnyl are often tried to help generalised fatigue. Psychological support should be offered to patients and caregivers, as patients face considerable uncertainty, may feel helpless during the course of their disease, and generalised anxiety is not uncommon. Patients and carers should be screened for frank depression and a combination of pharmaceutical and non-pharmaceutical interventions may be beneficial. Short course anxiolytics may be considered keeping the risk of dependency in mind. Antidepressants are usually prescribed more liberally, but side effects (hyponatraemia, sedation) and response to therapy need to be carefully monitored.

Endocrine and metabolic management

There is no evidence to support androgen replacement therapy in KD. The treatment of gynecomastia is surgical and a referral for a surgical consultation is typically only offered on the patient’s request. Insulin resistance and diabetes should trigger careful monitoring (HBa1C) and pharmacological treatment. The first line treatment of dyslipidaemia is dietary adjustments and statins should only be used judiciously. There is no established risk for additional myopathy associated with statin therapy in KD, but the risk/benefit ratio has to be assessed on a case-by-case basis depending on the severity of the dyslipidaemia and the vascular risk profile. Vitamin D deficiency needs rigorous substitution. Osteoporosis should be managed by current guidelines and pharmacological interventions are not KD-specific.

Rehabilitation and assistive devices

Physiotherapy should be initiated at the time of diagnosis to prevent complications related to limb weakness, limited range of motion and gait impairment. It has to be carefully tailored to individual disability, life-style, occupation, and fatigue. Physiotherapy can be beneficial in a home setting, in private practice, in a hospital, or in a specialist centre. Physiotherapy focuses on the maintenance of articular range of motion either by passive or active mobilisation and must be pain free, but is sometimes performed following analgesic administration or massages. Frequent pauses are offered during physiotherapy to prevent fatigue. Forms of ‘mechanotherapy,’ passive movements with motorised devices and motor electrostimulation with pulse generators are contraindicated. The aim of respiratory physiotherapy is the prevention of respiratory tract infections, bronchial obstruction, and phlegm mobilisation. These may include manual techniques, such as ‘breath stacking’ and instrumental interventions such as the use of cough assist machines (insufflator - exsufflator).

Speech therapy rehabilitation should be initiated at the onset of the first bulbar symptom. It should be tailored to the specific deficits of articulation, phonation or swallowing. Speech therapy entails both passive and active exercises to mobilise the relevant oropharyngeal muscles. Postures facilitating safe deglutition and prevent aspiration are also thought to patients and caregivers during the speech therapy sessions.

An expert occupational therapy assessment is required after KD is diagnosed. Interventions will be guided by individual preferences, life-style, home environment, driving needs, and working environment. Occupational therapy will focus on autonomy with regards to activities of daily living; mobilising, washing, dressing, feeding, writing, driving etc. To facilitate independence in routine daily activities assistive devices will be recommended to suit individual support needs. Occupational therapists typically recommend a range of aids and devices which can be tried before deciding which one to use. These typically include wheelchairs, electric chairs, mobility scooters, stair lifts, special keyboards, remote controls, raised toilet seats, taps with large handles, adapted kitchen utensils, large mobile phones etc. The occupational therapists will train the patient and caregivers how to use new aids safely and confidently. Home visits are important elements of individualised occupation therapy to evaluate home circumstances and consider optimal technical solutions for the patients specific needs; home automation, video bells, smart devices, chair lifts, walk-in showers, toilet adjustments, electric beds etc. The implementation of technical aids and home adaptations will be facilitated through an established infrastructure with reputable suppliers and fitters. Patient advocacy groups, charities and patient organisations often provide advice, information and funding regarding home modifications.

Nutritional management

Following a thorough nutritional assessment after the diagnosis is established; the dietician will provide expert advice on food supplements to facilitate adequate protein, energy and vitamin intake. For patients with dysphagia, consistency adjustments, texture changes, thickening powders, and gelled water may be recommended. Appropriate dental care is also indispensable to for satisfactory oral intake. Oral candidiasis is not uncommon due to the combination of atrophic tongue changes and insulin resistance, and careful mouth hygiene is required to prevent painful fissuring. Meticulous dental care, mouthwashes with antiseptics and antifungals are often recommended. Nutritional management must take the endocrine sequelae of the disease into account, especially dyslipidaemia or diabetes. Enteral nutrition, through gastrostomy may occasionally be needed and options include percutaneous endoscopic gastrostomy (PEG) or radiologically inserted gastrostomy (RIG) tubes. Main indications to consider enteral nutrition include: (1) unintentional weight loss greater than 10% of the baseline weight, (2) a (BMI) indicative of malnutrition (BMI < 18.5 18–70 years or BMI < 21 if older than 70), (3) considerable risk of aspiration pneumonia, (4) excessive time to finish meals impacting on social life. The risks and benefits of gastrostomy placement should be discussed with patients and caregivers, including the risk of local infections (cellulitis), need for tube changes, and the potential but remote risk of peritonitis. The practicalities of feeding should also be discussed with an experienced dietician, such as bolus versus overnight pump feeds, tube changes, the importance of tube flushing, cleaning the gastrostomy site and monitoring for infections, crushing medications or using syrup forms through the tube, and the option of continued oral intake even if a gastrostomy regime has been set up. In patients with respiratory compromise (RIG) may be preferable to PEG due to the lower risk of complications.

Respiratory management

Intermittent laryngospasm is not uncommon, reported in up to half of the patients. They can cause considerable anxiety, but they are relatively benign. Provoking factors such as gastroesophageal reflux should be aggressively treated with antacids. Respiratory management includes physiotherapy with drainage and positioning interventions. Insufflator - exsufflator devices (“cough assist” machine) may be needed to help the clearance of tracheal and bronchial secretions. Non-invasive mechanical ventilation is seldom required, and is best initiated after a joint neurological-respiratory consultation based on overnight pulse oximetry readings and early morning arterial blood gas analyses. Oxygen therapy should only be considered in a palliative management setting as without pressure support it may lead to reduced respiratory drive.

Cardiac management

Brugada syndrome can be identified based on a screening ECG, ambulatory ECG monitoring (Holter), signal averaged ECG, or invasive electrophysiological studies. It is imperative that the management of Brugada syndrome is coordinated by a cardiology team specialised in arrhythmia or cardiac electrophysiology. The risk for potentially life-threating ventricular arrhythmias, such as ventricular fibrillation (VF) or polymorphic ventricular tachycardia (PVT) needs to be stratified and implantable cardioverter defibrillator (ICD) devices should be considered. Preventive pharmacological interventions are less favourable, and quinidine is only prescribed in selected cases in patients who have ICDs. Life-style modifications should also be recommended in Brugada syndrome to decrease the risk for VF/PVT, such as avoiding strenuous exercise, excessive alcohol consumption, and certain medications such as high-dose paracetamol. For high-risk patients who had been defibrillated several times by their ICDs, radiofrequency catheter ablation is an invasive therapeutic option.

#### The follow-up of patients with KD

##### General objectives

The governing concepts of caring for patients with KD should include the following:

- Understating the practical implications of motor disability (life style restrictions, work, mobility).

- Screening for and foreseeing potential complications (chest infections, falls, depression).

- Responding dynamically to progressive changes.

- Initiating symptomatic treatments, supportive care, psychosocial care in a timely manner.

- Accompanying the patient and caregivers in their journey throughout the disease.

- Provide advice, information, support, education throughout the course of the disease.

- Linking in the patients with the relevant advocacy groups, charities, support groups.

- Offer genetic counselling for the patient and relatives.

##### Professionals involved

Patient follow-up is the joint responsibility of the general practitioner and neurologist and is ideally coordinated from a tertiary reference centre. KD care is fundamentally multidisciplinary, involving the general practitioner, neurologist, geneticist, endocrinologist, pulmonologist, cardiologist, rehabilitation specialists, nutritionist, ENT, specialist nurses, physiotherapist, occupational therapist, speech therapist, dietitian, clinical psychologists and social workers. Given the relatively slow evolution of the disease, the various specialists are involved in the patients care at different stages of the disease depending on the clinical symptoms.

##### Clinical follow-up

In the absence of distressing new symptoms, routine clinical follow-up is organised every six months to assess functional impairment, review symptomatic treatment (pain, cramps, cough etc.) and adjust rehabilitation strategies (physiotherapy, speech therapy etc.). Functional rating scales (SBMA-FRS) are not considered particularly sensitive to track subtle motor decline longitudinally. Routine follow-up reviews also provide an opportunity to screen for new extra-neurological manifestations, such as diabetes, genitourinary problems, metabolic disorders, cardiac disorders etc., serum creatinine is not optimal to screen for renal impairment as levels may remain in normal range despite decreased glomerular filtration due to concomitant muscle volume loss and poor dietary intake. Cystatin C measurements are superior to detect renal impairment as it is independent of muscle mass, gender, age, and there is no lag between kidney injury and cystatin rise [142]. Respiratory monitoring includes sitting and lying spirometry, measurement of maximal inspiratory and expiratory pressures (PI max and PE max), (SNIP) and peak cough flow measurements. If the patient is asymptomatic from a respiratory point of view, routine spirometry once a year may be judicious. If the maximum PI or SNIP is less than 60% of the normative reference value, arterial blood gas analyses are recommended. Polysomnography should also be performed if sleep apnoea is suspected or in case of unexplained fatigue and daytime somnolence. Weight should be rigorously monitored and recorded monthly. Fasting blood glucose, HbA1c, and the lipid profile should be checked annually. In case of established insulin resistance or frank diabetes, the relevant surveillance protocols will apply including screening for vasculopathic changes, skin surveys, renal and retinal screening etc. An urgent mammogram and/or ultrasound should be requested if there is any change in gynecomastia, especially if it is unilateral, or if an underlying mass is suspected. A routine ECG should also be recorded every year and carefully archived (in a chart or digitally) to enable comparisons with subsequent recordings. An initial osteodensimetry is recommended and if it is normal, it should only be repeated if there is an additional risk for osteoporosis.

## Discussion

While best practice recommendations and management guidelines have been repeatedly published for ALS in the US, Europe and Japan, no such guidelines exist for Kennedy’s disease despite similarities in diagnostic challenges, heterogeneity of manifestations and the wide spectrum of motor and extra-motor symptoms. Dedicate KD management guidelines not only raise awareness of a low-incidence neuromuscular condition, they draw attention to the range of extra-neurological manifestations associated with the condition and provide a framework to anticipate and screen for serious cardiac and complex endocrine manifestations. KD-specific best practice recommendations provide guidance to the complex supportive needs of the condition and emphasise the importance of well-timed multidisciplinary interventions. As with other progressive neurodegenerative conditions, the governing principle of KD care is the maintenance of independence, preservation of autonomy, dignity and prevention of serious medical complications. Given the low-incidence of KD and the multi-organ, multi-system involvement, best practice recommendations are best proposed by large consortia the members of which actively participate in the care of KD patients. The lack of effective disease-modifying therapies, absence of validated biomarkers, potentially life-threatening cardiac and respiratory complications and the paucity of clinical trials add to the urgency of publishing consensus management recommendations. While our recommendations cover the most stereotypical presentations and possible complications, considerable clinical variations exist which require flexible, dynamic multidisciplinary interventions especially in the presence of comorbid medical conditions. This protocol is therefore not exhaustive and merely provides a framework for KD management.

## Conclusions

In the context of an orphan disease, these guidelines provide a reference care pathway for patients with KD both for general practitioners and general neurologist who may be less familiar with the condition. This protocol will need to be regularly updated based on emerging scientific findings, especially therapeutic advances, such as gene therapy. There are important lessons to learn from international initiatives, such as the European Group for KD [10, 12]. Collaborative efforts through international consortia and multicentre registries are likely to contribute further to the characterisation of the natural history of the condition, the establishment of disease-specific biomarker panels and ultimately contribute to the development of effective disease-modifying therapies.

## Methods

### Protocol development

**Fig. 1:**
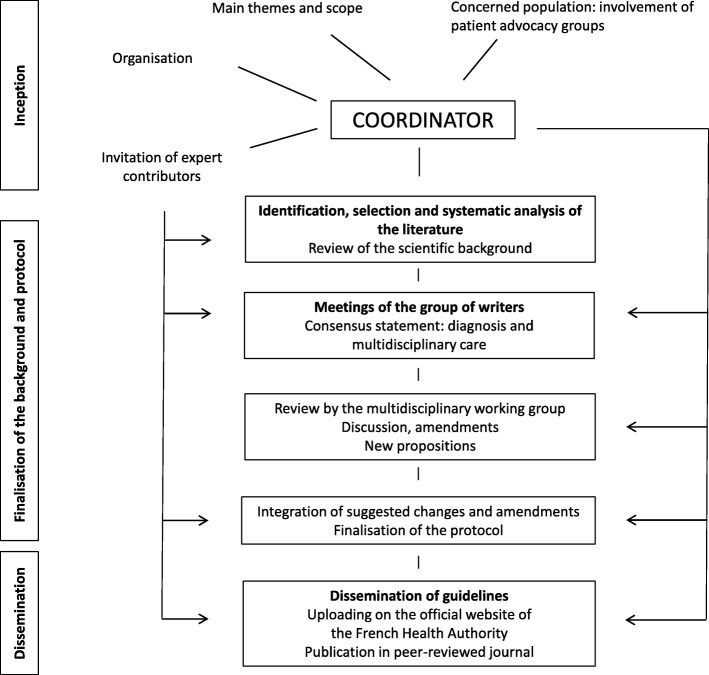
Development of the national Kennedy’s disease protocol

The protocol is based on currently available scientific evidence and provides a consensus statement for the care of KD patients (Fig. 1). The multi-centre working group included French specialists and therapists with experience in the diagnosis and management of KD, international experts, and representatives of the French Association for Research in ALS (ARSLA). The process of protocol development is outlined in Fig. 1. Contributors for each section were selected based on their clinical expertise in specific aspects of KD. Their names and affiliations are listed in the Supplementary material. Guideline development was coordinated by the first author and supported by a group of the multidisciplinary working group. The coordinator (first author) had the following responsabilities: 1) determining the scope of the protocol and the specific objectives; 2) defining core care requirements based on a systematic literature review; 3) identifying the main national reference centres involved in KD care; 4) inviting expert co-authors to the multidisciplinary working group; 5) establishing and maintaining a timeline for guideline development.

Two investigators (EB and PFP) have first performed an independent literature search. They reviewed the available international literature using standard databases (PubMed, MEDLINE, Cochrane Database of Systematic Reviews, EMBASE and Scopus) to evaluate evidence for the efficacy of specific interventions. They graded the level of evidence according to the French Health Authority guidelines [143] (Table 5). The pair of investigators then performed a systematic analysis of the literature which was circulated to the co-authors. Subsequently, the co-authors drafted an initial protocol version based on bibliographic research. The co-authors contributed to specific manuscript sections based on their sub-specialty and clinical expertise. Formal meetings were then organised by the coordinator to produce a consensus document. The multi-disciplinary working group of 19 members was tasked with the evaluation, correction or validation of the initial version of the protocol. All statements had to be unanimously agreed to by all members of the working group to be included in the final draft. The final protocol was submitted to the French Health Authority for validation and published on the agency’s website [144]. The French Health Authority committed to the dissemination of the protocol at relevant scientific platforms (website, publications, and presentations at meeting).

The working group decided to update the background section of the document which was provisionally finalised in March 2017 to include scientifically relevant new publications in the field. The updated scientific section was meticulously reviewed again and approved by the multidisciplinary group.

### Areas adressed

The list of the topics addressed is presented in Table 6.

**Table 6:** Specific themes reviewed and elaborated upon for the development of the French national KD protocol

Scientific background	KD Management Protocol
**I. Pathophysiology** **II. Epidemiology** **III. Natural history** **IV. Genotype-phenotype correlations** **V. The clinical spectrum of KD: Diagnostic considerations** **5–1) Neurological signs and symptoms** 5.1.1 Motor and sensory signs and symptoms 5.1.2 Neuropsychological deficits **5.2) Extra-neurological manifestations** 5.2.1 Endocrine abnormalities 5.2.2 Metabolic involvement 5.2.3 Bone involvement 5.2.4 Cardiac involvement 5.2.5 Genitourinary disorders **5.3) Laboratory examinations** 5.3.1 Muscle biopsy 5.3.2 Biology 5.3.3 Imaging **VI) Differential diagnoses** **VII) Management**	**I. Diagnosis and Evaluation** 1.1. Diagnosis and initial evaluation 1.2. Professionals involved 1.3. Clinical cues 1.4. Neurologic signs and symptoms 1.5. Extra-neurological signs and symptoms 1.6. Confirmation of the diagnosis 1.6.1. Electrophysiology (EMG/NCS) 1.6.2. Genetic confirmation 1.6.3. Differential diagnoses 1.7. The announcement of the diagnosis and patient information 1.8. Evaluation of severity of disease, screening for KD-associated comorbidities 1.9. The evaluation of neurological signs 1.10. Respiratory and nutritional assessment 1.11. Endocrine and metabolic evaluation 1.12. Cardiac evaluation 1.13. Bone health assessment 1.14. Genitourinary evaluation 1.15. Genetic counselling **II. Medical management** 2.1. General objectives 2.2. Professionals involved 2.3. Therapeutic management 2.3.1. pharmacological treatment 2.3.2. non-pharmacological interventions 2.4. Pharmacological treatments for pain 2.5. Treatment of fatigue and mood disorders 2.6. Endocrine and metabolic management 2.7. Rehabilitation 2.8. Nutritional management 2.9. Respiratory management 2.10 Cardiac management **III) Follow-up** 3.1. General objectives 3.2. The multidisciplinary team 3.3. Clinical follow-up 3.4. Health care professional follow-up

## Supplementary information

**Additional file 1.**

## Data Availability

The draft KD protocol is published in French on the French Health Authority’s website (https://www.has-sante.fr/jcms/c_2776017/fr/amyotrophie-bulbo-spinale-liee-a-l-x-ou-maladie-de-kennedy).
