# Supplementary material for: The French national protocol for Kennedy’s disease (SBMA): consensus diagnostic and management recommendations
Source: Orphanet J Rare Dis. 2020 Apr 10;15:90. doi: 10.1186/s13023-020-01366-z (PMC7149864; doi:10.1186/s13023-020-01366-z)
Supplement: Supplementary file 1 — Additional file 1. [file 13023_2020_1366_MOESM1_ESM.docx]

**SUPPLEMENTARY MATERIAL 1: names and affiliations of contributors**

**Coordinator**

Pierre-François Pradat (Neurologist, Tertiary Referral Center for ALS and Motor Neuron Diseases, Paris)

**Literature review**

Pierre-François Pradat (Neurologist, Tertiary Referral Center for ALS and Motor Neuron Diseases, Paris)

Emilien Bernard (Neurologist, Tertiary Referral Center for ALS and Motor Neuron Diseases, Lyon)

**Diagnostic and managment protocol**

1. **Neurologists**

Pierre-Francois Pradat **(**Neurologist, Paris)

Philippe Corcia (Neurologist, Tours)

François Salachas (Neurologist, Paris)

Christophe Vial (Neurologist, Lyon)

Emilien Bernard (Neurologist, Lyon)

Peter Bede (Neurologist, Paris)

Philippe Couratier (Neurologist, Limoges)

1. **Other physician specialists**

Capucine Morélot-Panzini (Respiratory physician, Paris)

Christel Jublanc (Endocrinologist, Paris)

Karim Whabi (Cardiologist, Paris)

**Multi-disciplinary working group**

1. **Professionals working at French tertiary referral centre for motor neuron diseases**
   1. **Neurologists**

Claude Desnuelle (Nice)

Nadine Le Forestier (Paris)

- 1. **Respiratory Physician**

Thierry Perez (Lille)

- 1. **Physiotherapist**

Cédric Ramos (Nice)

- 1. **Occupational therapists**

Sandrine Maniez (Paris)

Julie Robillard (Tours)

1. **Psychologist**

Christophe Coupe (Paris)

1. **Neuropsychologists**

Laurence Laurier Bertram (Lille)

Sandra Roy Bellina (Montpellier)

1. **Speech therapist**

Nathalie Lévêque (Paris)

1. **Social worker**

Jérôme Penot (Strasbourg)

1. **Other professionals involved in the care of KD patients**
   1. **Neurologist**

Andoni Echaniz-Laguna (Strasbourg)

- 1. **Geneticist**

Cyril Goizet (Bordeaux)

- 1. **Rehabilitation medicine physician**

Bertrand Pichon (Paris)

- 1. **Physician specialised in nutrition**

Jean Claude Desport (Limoges)

- 1. **Endocrinologist**

Michel Pugeat (Lyon)

1. **General Practitioner**

Valérie Goutines Caramel

1. **International experts**

Giorgia Querin (Neurologist, Italy)

Gianni Soraru (Neurologist, Italy)

**SUPPLEMENTARY MATERIAL 2**

CLINICAL SCALES USED IN KENNEDY’S DISEASE

**SBMA Functional Rating Scale (SBMA-FRS)**

**SBMA-FRS** (Reference: Hashizume A et al. Neuromuscular Disorders. 2015 (25): 554-562)[134].

| **Bulbar-related subscale** | |
| --- | --- |
| Speech | 4. Normal speech.  3. Speaks in a slightly nasal tone.  2. What is said can be understandable despite impaired speech.  1. A portion of what is said cannot be understandable unless heard over and over again.  0. Other media than voices are needed for communication. |
| Control of salivation | 4. No drooling.  3. Dribbles at night or during eating (less often than once a week).  2. Occasionally dribbles at night or during eating (not less often than once a week).  1. Dribbles on other occasions than above.  0. Dribbles so much that always needs tissues or a handkerchief. |
| Swallowing | 4. No difficulty.  3. Occasionally chokes on food depending on its texture.  2. Avoids some particular textures of food that may cause choking, etc.  1. Food textures have to be always modified to soft or chopped ones.  0. Enteral or intravenous nutrition is needed. |
| Tongue | 4. No atrophy.  3. Atrophic, but the tongue tip can be smoothly moved to and fro between the left and right corners of the mouth.  2. The tongue tip can be moved, but not smoothly, to and fro between the left and right corners of the mouth.  1. The tongue tip cannot reach the mouth corner.  0. No motion of the tongue. |
| Puffing cheeks | 4. The air does not leak when the cheeks are firmly pressed.  3. The air leaks when the cheeks are firmly pressed.  2. The cheeks can be puffed out, but not fully.  1. Although the lips can be pursed, the cheeks cannot be puffed.  0. The lips cannot be pursed. |
| **Upper limb-related subscale** | |
| Writing | 4. No difficulty.  3. Slightly difficult in writing, but can write a long sentence as well.  2. Writing gets slow in writing a long sentence.  1. Difficult in writing even a short sentence.  0. Unable to write letters. |
| Eating action | 4. No problem with eating actions.  3. Able to finish eating within a normal time, but has some  difficulty in using a knife and a fork.  2. Takes a longer eating time due to his difficulty in using a  knife and a fork.  1. Unable to use a knife or a fork, but good enough at using a spoon.  0. Needs assistance for eating. |
| **Trunk-related subscale** | |
| Dressing activity | 4. No problem.  3. Partially impaired, but taking no additional time to change  clothes.  2. Partially impaired, so takes time to change clothes because he needs to sit or lean against something.  1. Partial assistance is needed.  0. Total assistance is needed. |
| Rising from a sitting position | 4. No problem.  3. Minor problem.  2. Able to rise all alone, but needs to hold on something.  1. Any assistance is needed.  0. Unable to rise at all. |
| Arising from a  supine position | 4. No problem.  3. Minor problem.  2. Able to arise all alone, but needs some efforts, ex.) holding a lateral position beforehand.  1. Any assistance is needed to arise.  0. Unable to arise at all. |
| Bowing | 4. Able to bow his head fully in a standing position.  3. Able to bow his head in a standing position, but not fully.  2. Able to hold a standing position, but slightly hard to bow his head.  1. Difficult in holding a standing position, but able to bow his  head in a sitting position.  0. Unable to bow his head even in a sitting position. |
| **Lower limb-related subscale** | |
| Walking | 4. No problem with walking or running.  3. No problem with walking, but it is difficult to run.  2. A cane or some other assistive device is sometimes needed.  1. A cane or some other assistive device is always needed.  0. Walking is impossible. |
| Stairs | 4. No problem with walking upstairs.  3. Able to walk upstairs without a handrail, but slowly.  2. Able to walk upstairs without assistance if a handrail is  available.  1. Assistance from someone is needed.  0. Unable to walk upstairs anyhow. |
| **Breathing-related subscale** | |
| Breathing | 4. No dyspnea  3. Gets short of breath during activity like walking.  2. Gets short of breath even during slight activity like standing up.  1. Has shortness of breath even at rest.  0. A respiratory assistor such as NIPPV and TPPV is needed. |

**Medical Research Concil (MRC: Testing)**

1. No movement
2. Flicker of movement
3. Through full range actively with gravity counterbalanced
4. Through full range actively against gravity
5. Through full range actively against some resistance
6. Through full range actively against strong resistance
